# Paired-Pulse Parietal-Motor Stimulation Differentially Modulates Corticospinal Excitability across Hemispheres When Combined with Prism Adaptation

**DOI:** 10.1155/2016/5716179

**Published:** 2016-06-22

**Authors:** Selene Schintu, Elisa Martín-Arévalo, Michael Vesia, Yves Rossetti, Romeo Salemme, Laure Pisella, Alessandro Farnè, Karen T. Reilly

**Affiliations:** ^1^Integrative Multisensory Perception Action & Cognition Team (ImpAct), INSERM U1028, CNRS UMR5292, Lyon Neuroscience Research Center (CRNL), 69000 Lyon, France; ^2^University of Lyon 1, 69000 Lyon, France; ^3^Krembil Research Institute, Toronto Western Hospital, University Health Network, Toronto, ON, Canada M5T 2S8; ^4^Hospices Civils de Lyon, Neuro-Immersion & Mouvement et Handicap, 69000 Lyon, France

## Abstract

Rightward prism adaptation ameliorates neglect symptoms while leftward prism adaptation (LPA) induces neglect-like biases in healthy individuals. Similarly, inhibitory repetitive transcranial magnetic stimulation (rTMS) on the right posterior parietal cortex (PPC) induces neglect-like behavior, whereas on the left PPC it ameliorates neglect symptoms and normalizes hyperexcitability of left hemisphere parietal-motor (PPC-M1) connectivity. Based on this analogy we hypothesized that LPA increases PPC-M1 excitability in the left hemisphere and decreases it in the right one. In an attempt to shed some light on the mechanisms underlying LPA's effects on cognition, we investigated this hypothesis in healthy individuals measuring PPC-M1 excitability with dual-site paired-pulse TMS (ppTMS). We found a left hemisphere increase and a right hemisphere decrease in the amplitude of motor evoked potentials elicited by paired as well as single pulses on M1. While this could indicate that LPA biases interhemispheric connectivity, it contradicts previous evidence that M1-only MEPs are unchanged after LPA. A control experiment showed that input-output curves were not affected by LPA* per se*. We conclude that LPA combined with ppTMS on PPC-M1 differentially alters the excitability of the left and right M1.

## 1. Introduction

Unilateral spatial neglect frequently occurs after right hemisphere damage and is an invalidating multicomponent syndrome in which perception of the contralesional side of space is compromised [1, 2]. In line with the hemispheric rivalry theory [3], neglect patients bias their attention toward the right side of space [4], likely resulting from hemispheric imbalances in cortical excitability. Specifically, damage to the right hemisphere (RH) decreases activity in the contralesional cortex, which then leads to an increase in the excitability of the intact left hemisphere (LH) through the release of interhemispheric inhibition [5, 6]. Recent evidence suggests that the hyperexcitability of parietal-motor (PPC-M1) functional connections in the intact LH of patients with neglect can be reduced by applying inhibitory repetitive transcranial magnetic stimulation (rTMS) to the left posterior parietal cortex (PPC). Interestingly, these neurophysiological changes were associated with a decrease in the severity of neglect symptoms [7, 8].

Inhibitory rTMS applied to the RH of healthy individuals can induce neglect-like biases, such as a rightward shift in line bisection judgments [9–11]. Neglect-like biases in line bisection and attention can also be elicited with a simple sensorimotor procedure, such as adaptation to leftward shifting prisms (LPA) [12–14]. Prism adaptation is a sensorimotor process that induces plastic changes in the brain by altering both sensory-motor correspondences and visuospatial cognition [15–18]. Remarkably, while rightward prism adaptation (RPA) decreases the severity of neglect symptoms in patients [16, 19, 20], in healthy individuals, LPA induces a rightward shift on tasks that measure visuospatial and temporal cognition [14, 21]. Accordingly, it is often described as inducing a neglect-like bias and is frequently used as a “model” of neglect. Critically, while there are similarities in the direction of the attentional bias observed in patients after RH damage and in healthy individuals after LPA, to date there is no evidence for similar physiological changes associated with these biases.

One possible explanation for the mechanism of action of LPA is that it parallels that of a right hemisphere stroke: LPA decreases excitability in the right PPC and alters left PPC excitability through changes in interhemispheric inhibition [22]. However, no study to date has measured LPA-induced changes in PPC-M1 activity and related behavioural performance. Since PPC-M1 functional connections in the intact left hemisphere of neglect patients appear to be hyperexcitable [7], the aim of this study was to test whether the neglect-like behavior induced by LPA in healthy individuals is accompanied by excitability changes in PPC-M1 functional connections.

To investigate parietal-motor changes in both the left and right hemispheres we used a dual-site paired-pulse protocol used elsewhere [23, 24], which quantifies the influence of PPC over M1 and thus provides an index of the strength of parietofrontal functional connectivity within a hemisphere. Since inhibitory rTMS over the right parietal cortex induces neglect-like behavior in line bisection judgments in healthy individuals [9], we hypothesized that LPA would modulate the strength of PPC-M1 interactions by decreasing the excitability of the right parietal cortex and increasing that of the left.

## 2. Materials and Methods

### 2.1. Participants

Twenty-eight healthy volunteers (14 males, mean age = 25.14 years, SEM = 0.72) participated in Experiment  1. Ten healthy volunteers (8 females, mean age of 28.4 years, SEM = 2.31) participated in Experiment  2. All participants had normal or corrected-to-normal vision, were right-handed according to the Edinburgh Handedness Inventory [25], gave written informed consent, and received payment for their participation in the study. The study was approved by the local ethics committee and was conducted in accordance with the ethical standards of the 1964 Declaration of Helsinki (last update: Seoul, 2008).

### 2.2. Experimental Procedure

#### 2.2.1. Experiment 1: Dual-Site Paired-Pulse TMS to Measure PPC-M1 Functional Connections

All participants were adapted to prisms that shifted the visual field 15 degrees to the left (LPA) and PPC-M1 connections were measured before and after LPA in either the left (*n* = 14, 7 males, mean age = 25.5 years, SEM = 1.12) or right hemisphere (*n* = 14, 8 males, mean age = 24.78 years, SEM = 0.95).

As a measure of the efficacy of LPA in inducing rightward visuospatial bias in bisection we used a Landmark task [26]. In this task, most healthy individuals show a bias termed “pseudoneglect” [27], which is the tendency to perceive the line's center slightly to the left of its true center. At the population level, however, this bias is variable (see [28] for a review), and possibly depends on individual differences in the strengths of the frontoparietal attention network, which is known to predict performance on line bisection tasks [29, 30]. The presence or absence of pseudoneglect not only may reflect two different observer types [31] but also might influence PA-induced modulations in line bisection tasks [32]. Therefore, we decided to reduce the variability in our population by studying PPC-M1 interactions in a pseudoneglect population.

This between-subjects design experiment consisted of a perceptual line bisection task (Landmark), paired-pulse TMS (ppTMS), and open-loop pointing measures both before and after LPA. The order of administration of the Landmark task and ppTMS was counterbalanced across participants, while the timing of the open-loop measurements was kept constant. Throughout the experiment participants were comfortably seated on an armchair with their head positioned on a neck-rest during the ppTMS and on a chinrest during the LPA, Landmark task, and open-loop pointing tasks. In order to reduce the possibility of deadaptation, after LPA, participants were instructed to keep their eyes closed and avoid moving their right hand during the short intervals between tasks.

*Open-Loop Pointing*. Participants were seated in front of a white horizontal board on which three target dots (5 mm diameter) were positioned at 0, −10, and +10 degrees from their body midline with the central point 57 cm from their nasion when their head was positioned in the chinrest. Participants performed six pointing movements to the central target (0°) without visual feedback. Before each of the six pointing movements they were instructed to look at the central target (0°), close their eyes, point to the target with their right index finger while keeping their eyes closed, and then return their hand to the starting position. The delay between participants closing their eyes and pointing to the central target ranged between 1 and 2 seconds. To ensure that participants had no visual feedback regarding either their movement or their landing position, vision of the hand was occluded before onset of the pointing movement. Open-loop pointing performance was calculated as the average of the six pointing movements to the central target.

*Landmark Task*. A computer screen was positioned in front of the participants who kept their head on the chinrest. A series of prebisected lines appeared on the screen and participants were instructed to fully inspect each line and judge whether the mark (transector) was closer to the left or the right end of the line. In this two-alternative forced-choice paradigm participants were instructed to respond accurately and quickly by pressing a pedal with their left foot if the transector was perceived as being closer to the left end of the line or with their right foot if they thought it was closer to the right end. Prior to the first administration of this task at least ten practice trials were given to ensure that participants properly understood the instructions and were comfortable answering with the pedals.

Stimuli were white lines (350 mm × ~2 mm) displayed on a black screen positioned 35 cm from the participant's eyes. Lines were transected at the true center and 2, 4, 6, 8, and 10 mm toward either the left or right side of the true center. Each of the 11 different prebisected lines was presented six times in a random order, yielding a total of 66 trials, which took approximately three minutes to complete. Each prebisected line was displayed for a maximum of five seconds or until a response was made and was then replaced by a black-and-white patterned mask, which stayed on the screen for one second before the next prebisected line was displayed. Presentation software (Neurobehavioral Systems, Inc., USA) was used to generate the stimuli, record responses, and control the timing of stimulus presentation throughout the task. For each participant the percentage of “right” responses was plotted as a function of the position of the transector. These data were then fitted with a sigmoid function and the value on *x*-axis corresponding to the point at which the participant responded “right” 50% of the time was taken as that participant's point of subjective equality (PSE).

*Prism Adaptation*. Participants faced the white horizontal board wearing prismatic goggles that deviated their visual field leftward by 15 degrees. They performed a total of 150 verbally instructed pointing movements with their right index finger towards the right (+10°) and left (−10°) targets in a pseudorandom order. Before pointing, they placed their right index finger on the starting position. Participants could not see their hand when it was in the starting position or during the first third of the pointing movement. They were instructed to point with the index finger extended, to execute a one-shot movement at a fast but comfortable speed, and then to return their hand to the starting position. After 150 pointing movements the prismatic goggles were removed and the ppTMS, Landmark, and open-loop pointing measurements were repeated.

*Electromyogram Recording*. Electromyographic (EMG) recordings were made from the left (RH-TMS) or right (LH-TMS) first dorsal interosseous (FDI) muscles using Ag-AgCl surface electrodes (Delsys). EMG activity was sampled at 2000 KHz, digitalized using an analogue-to-digital converter (Power 1401II, Cambridge Electronics Design, Cambridge, UK), and stored on a personal computer for off-line data analysis using SIGNAL software (Cambridge Electronic Devices, Cambridge, UK).

*TMS Procedure*. A paired-pulse transcranial magnetic stimulation (ppTMS) approach using two coils was used to deliver pulses either to M1 alone (test pulse: TS) or to M1 after a pulse was delivered to the ipsilateral PPC (conditioning pulse: CS). TMS pulses were delivered using two custom-made branding iron figure-of-eight coils (external diameter: 50 mm) each connected to a 200^2^ monophasic stimulator (The Magstim Company, Carmarthenshire, Wales), which operated as two independent stimulators. The coil over M1 was held tangentially to the scalp at an angle of approximately 45 degrees from the midline [33], while the other coil was positioned over the ipsilateral parietal cortex, P3 (left hemisphere group) or P4 (right hemisphere group) according to the 10–20 EEG system, and was rotated approximately 10° medially in order to induce a posterior-to-anterior directed current in the underlying cortical tissue [23]. Neuronavigation (Brainsight, Rogue Research) was used to monitor the position of both coils throughout the experiment and to ensure accurate coil repositioning after LPA. In one group of participants TMS was applied on the left hemisphere (LH-TMS group), whereas in the other group it was applied on the right hemisphere (RH-TMS group).

Participants rested their arm on a pillow placed on their lap or on the armrest of the chair and leaned their head on a neck-rest. After identifying the hotspot, the scalp location where stimulation evoked the largest MEP from the contralateral FDI muscle, we determined the resting motor threshold (RMT), defined as the lowest stimulation intensity that evoked at least five out of ten MEPs of at least 50 *μ*V peak-to-peak amplitude [34]. We then determined the stimulation intensity necessary to evoke an average MEP of approximately 1 mV (from 10 MEPs) in the relaxed FDI. This was then set as the TS intensity, while 90% of the resting motor threshold was used as the CS intensity.

The interstimulus intervals (ISIs) between the CS and TS were 2, 4, 6, and 8 ms [35]. Twenty TS-only trials and twelve CS-TS trials for each ISI were delivered in a fully random order, with intertrial intervals between 5 and 7 seconds. During the experiment we monitored MEP amplitude by eye. When the MEP amplitude was less than 50 *μ*V or the trial was clearly contaminated by muscular contraction we delivered extra trials in an attempt to have 20 valid TS-only and 68 valid PPC-M1 (12 per ISI) for each ppTMS session, which lasted between 9 and 11 minutes.

#### 2.2.2. Experiment 2: M1 Input/Output Curves to Measure Corticospinal Excitability

As in Experiment  1, throughout the experiment, participants were comfortably seated in an armchair with their head positioned on a neck-rest during the TMS and on a chinrest during the LPA and open-loop pointing tasks. The experimental protocol, this time a within-subjects design, consisted of measuring input/output (I/O) curves for each hemisphere and open-loop pointing measures both before and after LPA. The order of I/O curve measurements was counterbalanced across participants (i.e., right or left hemisphere first). The open-loop pointing task and LPA procedures were identical to Experiment  1.

*Electromyogram Recording*. EMG recordings were made from the left (LH-TMS) or right (RH-TMS) FDI. Electrode placement and EMG recording were identical to Experiment  1.

*TMS Procedure*. TMS pulses were delivered using a figure-of-eight coil (external diameter: 90 mm) connected to a Magstim 200^2^ magnetic monophasic stimulator (The Magstim Company, Carmarthenshire, Wales). The coil was held tangentially to the scalp over M1 at an angle of approximately 45 degrees from the midline [33]. Neuronavigation (Brainsight, Rogue Research) was used to monitor the position of the coil throughout the experiment and to ensure accurate coil repositioning after LPA.

Participants rested their arm on a pillow placed on their lap or on the armrest of the chair and leaned their head on a neck-rest. As in Experiment  1, after identifying the FDI hotspot, we determined the resting motor threshold (RMT). Both hotspot and RMT were measured for each hemisphere with the order counterbalanced across participants (i.e., either right or left hemisphere first).

I/O curves were measured by stimulating the left or right M1 (hotspot) twelve times at each of six different intensities, ranging from 90% to 140% of RMT in 10% steps. TMS pulses were delivered in a pseudorandom order, with intertrial intervals between 6 and 10 seconds. Each I/O curve was constructed using data from seventy-two stimulation pulses and the time to acquire the two curves was approximately 25 minutes. Individual participant I/O curves were calculated by averaging the peak-to-peak MEP amplitudes from all trials for a given intensity.

## 3. Results

### 3.1. Experiment 1: Dual-Site ppTMS Excitability to Measure PPC-M1 Functional Connections

**Figure 1:**
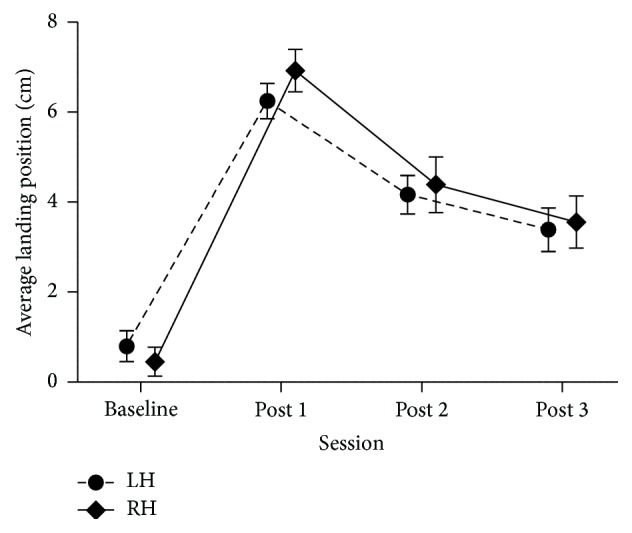
Average landing position for the open-loop pointing measure at baseline, post 1 (immediate after LPA), post 2 (20 minutes after LPA), and post 3 (45 minutes after PA) (*n* = 14 for each stimulated hemisphere group). Average landing position (*y*-axis) is represented in centimeters and bars represent standard errors of the mean (SEM).

*Open-Loop Pointing and Landmark Task*. To determine whether participants were significantly adapted immediately after LPA (post 1), after the first ppTMS session (post 2), and at the end of our experiment (~40 minutes after LPA, post 3) we performed a mixed-design ANOVA on the average landing position for the 6 open-loop pointing movements with Hemisphere stimulated (LH; RH) as a between-subject variable and Session (pre; post 1; post 2; post 3) as a within-subject variable. This analysis revealed a main effect of Session [*F*(3,78) = 148.43, *p* < 0.001], no main effect of Hemisphere [*F*(1,26) = .10, *p* = 0.752] nor an interaction between Session and Hemisphere [*F*(3,78) = 1.06, *p* = 0.369] (Figure 1). The LSD post hoc tests revealed that the landing position at all three evaluations after PA was significantly different from that before PA (0.6 cm) (*p*s < 0.001), and that each post-PA measurement was significantly different from the previous one (*p*s ≤ 0.006). This indicated that the whole group showed significant sensorimotor adaptation until the end of the experiment even though the amount of adaptation decayed significantly between each post-PA measurement.

**Figure 2:**
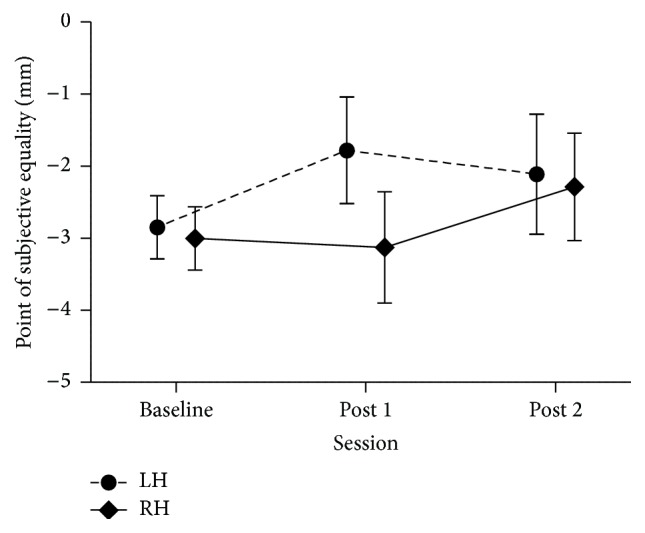
Average point of subjective equality (PSE) measured at baseline, post 1, and post 2 for each stimulated hemisphere group. PSE (*y*-axis) is represented in millimeters and error bars represent standard error of the mean (SEM).

As expected, the average point of subjective equality (PSE) of our preselected pseudoneglect participants was to the left of zero before PA (mean −2.9 mm, SEM 0.3 mm). To determine whether participants shifted their midline judgments after LPA we performed a mixed-design ANOVA on the point of subjective equality with Session (pre; post 1; post 2) as a within-subject variable and Hemisphere stimulated (LH; RH) as a between-subject variable. This analysis revealed no main effects or interactions (Session [*F*(2,52) = 1.79, *p* = 0.177], Hemisphere [*F*(1,26) = .43, *p* = 0.519], and Session × Hemisphere [*F*(2,52) = 1.54, *p* = 0.224]).  Figure 2 shows, however, that both groups tended to shift their midline judgments rightward (as expected after LPA), with the left hemisphere group shifting in the first evaluation after PA (1.1 mm) and remaining shifted (0.7 mm) at the second evaluation and the right hemisphere group shifting at the second (0.7 mm) but not the first evaluation (0.1 mm).

In conclusion, although our behavioral data revealed a tendency for subjects to shift their perceptual line bisection judgment rightward, this largely expected trend did not reach significance. Moreover, the group stimulated on the right hemisphere showed this trend only at post 2.

**Figure 3:**
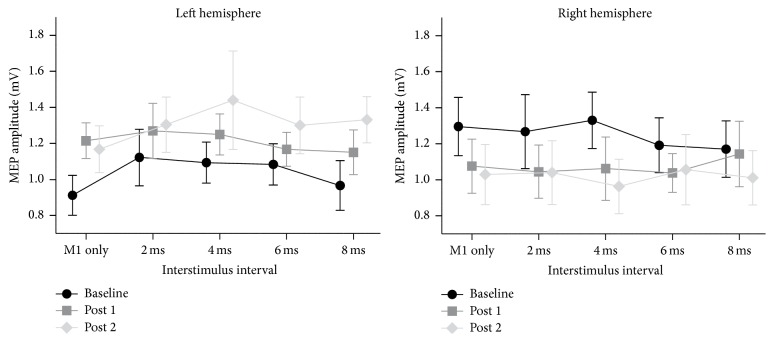
Raw MEP amplitudes shown separately for the five different stimulation conditions (M1-only and 4 PPC-M1 ISIs) at baseline, post 1, and post 2 for each stimulated hemisphere. MEP amplitude (*y*-axis) is represented in millivolts (mV) and error bars represent standard error of the mean (SEM).

*ppTMS*. To assess the functional connectivity between PPC and M1 in each hemisphere before LPA we performed a mixed-design ANOVA with Hemisphere stimulated (left; right) as a between-subject variable and ISI (M1-only; 2; 4; 6; 8 ms) as a within-subject variable. This analysis revealed no main effects and no interactions (ISI [*F*(4,104) = 1.44, *p* = 0.225], Hemisphere [*F*(1,26) = 1.28, *p* = 0.269], and ISI × Hemisphere [*F*(4,104) = 1.13, *p* = 0.345]) (Figure 3).

To assess the functional connectivity between PPC and M1 before and after LPA, that is, to measure PA-induced changes in functional connectivity, we performed a mixed-design ANOVA with Hemisphere stimulated (left; right) as a between-subject variable and Session (Pre; Post 1; Post 2) and ISI (M1-only; 2; 4; 6; 8) as within-subject variables. This analysis revealed a significant interaction between Session and Hemisphere [*F*(2,52) = 5.25, *p* = 0.008] but no other main effects or interactions (all *p*s > 0.363). The LSD post hoc tests revealed that between pre and post 2 MEP amplitudes increased significantly in the LH group (*p* = 0.019) and decreased significantly in the RH group (*p* = 0.046) (Figure 3).

Since there was no main effect of ISI or any interaction between ISI and any other variables this result suggests that MEP amplitudes changed across all stimulation conditions (M1-only stimulation and PPC-M1 stimulations). Since all 5 levels contain a contribution from the M1-only stimulation, we performed a separate analysis on MEP amplitudes from the M1-only condition to investigate its contribution to the interaction observed above. The mixed-design ANOVA with Hemisphere stimulated (left; right) as a between-subject variable and Session (pre; post 1; post 2) as a within-subject variable revealed no main effects of Session or Hemisphere (both *p*s > 0.833) but a significant Session × Hemisphere interaction (*F*(2,52) = 5.72, *p* = 0.006). LSD post hoc tests revealed no difference between the LH and RH groups at baseline (*p* = 0.057), while between pre and post 1 and pre and post 2 MEP amplitudes increased significantly in the LH group (both *p*s < 0.048), and between pre and post 2 they decreased significantly in the RH group (*p* = 0.039) (Figure 3).

The significant change in the absolute excitability of the motor cortex, increase in LH and decrease in RH M1-only MEP amplitudes, suggests that the general increase we observed in raw MEP amplitudes across all five tested conditions (M1-only and 2, 4, 6, and 8 ms CS-TS ISIs) might have been triggered by M1 excitability changes. While this could indicate that LPA creates an imbalance between the cerebral hemispheres (similar to that created by a lesion of the right hemisphere), this finding is inconsistent with a recent report showing that M1-only MEPs are unchanged following PA [36].

Increases or decreases in the slope of motor cortex input/output curves provide a good measure of changes in corticospinal excitability (CSE). Thus, to further investigate possible LPA-induced changes in CSE we conducted a second experiment in which we recorded M1 input-output curves from both hemispheres of 10 subjects (none of whom were tested in Experiment  1) before and after adaptation to the same 15-degree leftward deviating prisms used in Experiment  1.

### 3.2. Experiment 2: M1 Input/Output Curves to Measure Corticospinal Excitability

*Open-Loop Pointing*. Participants pointed on average 1.2 cm to the right of the central target in the baseline measure (pre). To determine whether participants were significantly adapted immediately after LPA (post 1) and at the end of the experiment (30 minutes after LPA, post 2) we performed a one-way repeated measures ANOVA on the average landing position of the 6 open-loop pointing movements with Session (pre; post 1; post 2) as the within-subject variable. The analysis revealed a significant effect of Session (*F*(2,18) = 94.56, *p* < 0.001). LSD post hoc tests revealed that the landing position at both post 1 (6 cm) and post 2 (3.5 cm) was significantly different from that measured before LPA (1.2 cm); post 1 also differed from post 2 (all *p*s < 0.001). Thus, participants showed a significant sensorimotor adaptation until the end of the experiment, with a significant decay from the immediate (post 1) to the late (post 2) measurement.

**Figure 4:**
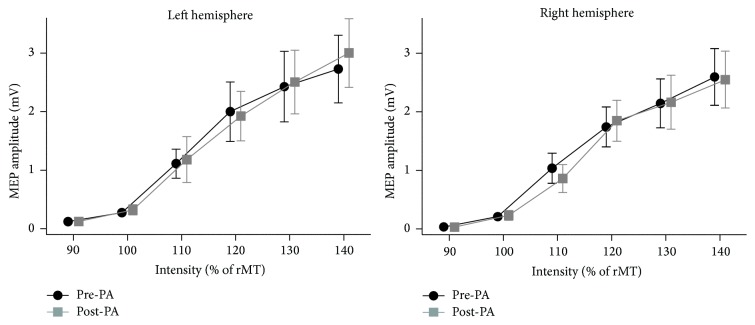
Input/output curve plots of average MEP amplitude across all subjects in Experiment  2 for both hemispheres (*n* = 10). MEP amplitude (*y*-axis) is represented in mV and error bars represent standard errors of the mean (SEM).

*I/O Curves*. To examine whether CSE was altered by LPA we conducted a repeated measures ANOVA on mean MEP amplitudes with Session (pre; post), Hemisphere (Left; Right), and Intensity level (6 levels; from 90% to 140% of rMT) as within-subject variables. Only the main effect of Intensity was significant (*F*(5,54) = 23.21, *p* < 0.0001) (Figure 4). None of the other main effects or interactions reached significance (all *p*s > 0.32).

To obtain the slope (and *R* ^2^) of each individual's curves [37, 38], I/O curves were fitted with linear regressions through the four middle stimulus intensities (100%, 110%, 120%, and 130%). Consistent with the absence of changes in MEP amplitudes shown in Figure 4, the two-way repeated measures ANOVA (variables Hemisphere stimulated and Session) on the slope of the best-fitting straight line through the middle four stimulus intensities did not reveal any significant differences in slope before and after LPA (all *p*s > 0.51).

Finally, for each participant we used the I/O curves to estimate (to the nearest 10% of RMT) the percentage of stimulator output required to produce a MEP of approximately 1 mV before and after LPA. We then performed a two-way repeated measures ANOVA (variables Hemisphere stimulated and Session) on these percentages. This revealed no significant main effects or interactions (all *p*s ≥ 0.42).

## 4. Discussion

The aim of this study was to investigate the physiological counterpart of the neglect-like behavior induced by LPA in healthy individuals. Our hypothesis was that neglect-like behavior arises from modulation of the strength of PPC-M1 interactions. Specifically, we hypothesized that LPA would decrease the activation of the right PPC, measured as a decrease in right hemisphere PPC-M1 connectivity, and that via the release of interhemispheric inhibition this would in turn increase activity in the left PPC, measured as an increase in left hemisphere PPC-M1 connectivity. Our findings provide only partial support for this hypothesis, as, at the behavioral level, the expected rightward shift was almost absent following LPA, particularly after parietal-motor ppTMS of the RH. At the neurophysiological level an asymmetrical modulation of MEPs in the predicted direction was observed (increase in the left hemisphere and decrease in the right one), but this was accompanied by a similar change when M1 was stimulated alone, thus casting some doubts on the specificity of this effect. Given that our control experiment ruled out the possibility that absolute CSE was modulated solely by LPA, we discuss the behavioral and neurophysiological results in the context of previous work, and we conclude by suggesting that the combination of LPA and parietal-motor ppTMS stimulation is responsible for differentially altering the excitability of the motor cortex in each hemisphere.

On the behavioral side, the open-loop pointing measure revealed that following LPA participants had a significant rightward visuomotor after effect. Surprisingly, despite the well-documented rightward shift in line bisection judgments after LPA (see, e.g., [13, 14]), we observed a tendency for subjects to shift their judgments rightward, but this was not significant. Furthermore, while LPA normally induces a rightward shift in the 10 to 35 minutes following the end of the adaptation procedure [14], participants stimulated on the right hemisphere showed no shift at the first measurement after LPA and showed a nonsignificant shift at the second measurement, that is, more than 20 minutes after prismatic adaptation.

On the neurophysiological side, MEP amplitudes were significantly modulated after LPA, increasing in the LH and decreasing in the RH. Importantly, this modulation was observed for both M1-only and PPC-M1 trials. Our analysis of CSE (M1-only trials) before and after LPA revealed that CSE increased in the LH and decreased in the RH. Since MEP amplitudes recorded on paired-pulse stimulation trials are inevitably affected by CSE, we cannot rule out the possibility that the PPC-M1 connectivity modulations we observed are mainly due to changes in CSE. In this respect, the left hemisphere increase and right hemisphere decrease in CSE could be interpreted as evidence in support of Pisella and colleagues' [22] hypothesis that LPA might create an imbalance between the two hemispheres by altering the excitability of the parietal cortices. Evidence that changing the excitability of the parietal cortex can affect CSE comes from a recent study showing that transcranial direct current stimulation (tDCS) over left PPC reduced the amplitude of left M1 MEPs after cathodal stimulation but increased left M1 MEPs after anodal stimulation [39]. We think this interpretation of our results is unlikely, however, as we observed no change in M1 input-output curves after LPA in our second experiment, a finding consistent with the results of Magnani and colleagues [36] who showed that PA does not alter CSE in either hemisphere, even though it produces an increase in intracortical facilitation in the hemisphere ipsilateral to the direction of the prismatic deviation.

An alternative interpretation is that the paired PPC-M1 stimulation could have induced corticocortical associative plasticity, as it has recently been shown [40] that paired associative stimulation (PAS) of the posterior parietal and motor cortices can induce associative plasticity. In this protocol, however, the delay between stimulating the two sites was crucially always fixed (5 or 8 ms depending on the protocol), as was the timing between subsequent pairs of pulses (5 seconds), whereas in our protocol there were four different delays between the parietal and motor stimuli (between 2 and 8 ms) and these 48 paired-pulse trials were intermingled with 20 M1-only pulses. Furthermore, the time between paired and single pulses varied between 5 and 7 seconds. Since PAS modulations in cortical excitability require strict time-dependent conditions [41–45], it seems unlikely that our paired-pulse stimulation alone could have induced a PAS-like modulation of CSE. Another argument against the possibility that our TMS protocol induced associative plasticity is the fact that the results of Koch et al. [40] would predict a change in left hemisphere CSE in the direction opposite to what we observe (i.e., a decrease in left M1 excitability). Instead, if we assume that LPA puts the two hemispheres into different excitability “states,” it is conceivable that the stimulation we used to measure parietal-motor interactions somehow interacted with the “state” of each hemisphere to differentially alter CSE.

While there is currently no evidence to suggest that paired stimulation of the type used here (i.e., no strict timing between parietal and motor stimuli or between subsequent stimuli pairs) can produce neuromodulatory effects, recent studies have demonstrated that MEP amplitudes are time variant [45] and a single TMS pulse over the motor cortex can induce cumulative changes in neural activity which may alter CSE [46]. Of particular importance to our finding is the observation by Pellicciari and colleagues [46] that CSE changes were present when single TMS pulses were delivered over M1 at either fixed (4 sec) or random (2.2–5.5 sec) interstimulus intervals. However, short interstimulus intervals appear to be critical to produce this effect, as a previous study showed that repetitive stimulation of M1 with an interstimulus interval of 10 seconds did not affect the amplitudes of subsequent MEPs [47].

The ppTMS technique has been widely used in recent years as a method to assess parietal-motor functional connectivity. Since our primary goal was to investigate the effects of prismatic adaptation, we chose to thoroughly examine the influence of prismatic adaptation on CSE rather than to look at the possible effect of multiple ppTMS measurements. While there is no published data directly investigating changes in CSE induced by multiple ppTMS measures, there is some evidence that repeated ppTMS measures do not alter CSE. For example, some studies include multiple measures because of their experimental design, and when MEP amplitudes for M1-only stimuli are reported (and the intensity of the stimulator was not adjusted to keep the test stimulus at an average amplitude of 1 millivolt [39]), it appears that multiple measures do not affect CSE [7, 23, 35, 48–50]. This, together with our own data showing no change in CSE in either hemisphere when LPA was applied alone, provides converging evidence in support of our conclusion that it is the combination of the ppTMS and LPA procedures that is at the origin of the opposite changes in M1 excitability observed in the two hemispheres. It is worth noting that the possibility that the combination of LPA with our parietal-motor stimulation paradigm had a neuromodulatory effect is supported by the absence of a significant behavioral shift in line bisection judgments. Indeed, despite testing a pseudoneglect population, which was presumably homogeneous with respect to anatomical features, we did not observe the significant rightward shift on line bisection judgments that is typically reported after LPA in similarly sized samples [13, 14].

To conclude, we designed a dual-site paired-pulse experiment with the intention of using parietal-motor interactions as a proxy for changes in parietal cortex excitability following adaptation to LPA. The differential change in CSE in the left and right hemispheres we observed, plus the absence of the well-documented right shift in line bisection judgments, leads us to suggest that LPA interacted with our parietal and motor cortex stimulation. We conclude, therefore, that under the physiological conditions produced by prism adaptation paired parietal-motor stimulation can act as a neuromodulator.
